# Surgical Treatment of Congenital True Macroglossia

**DOI:** 10.1155/2013/489194

**Published:** 2013-12-05

**Authors:** Sabrina Araújo Pinho Costa, Mário César Pereira Brinhole, Rogério Almeida da Silva, Daniel Hacomar dos Santos, Mayko Naruhito Tanabe

**Affiliations:** Department of Oral and Maxillofacial Surgery, Vila Penteado General Hospital, Avenue Ministro Petrônio Portela, 1642, Freguesia do Ó, 02802-120 São Paulo, SP, Brazil

## Abstract

Macroglossia is a morphological and volumetric alteration of the tongue, caused by muscular hypertrophy, vascular malformation, metabolic diseases, and idiopathic causes and also associated with Down and Beckwith-Wiedemann syndromes. This alteration can cause dental-muscle-skeletal deformities, orthodontic instability, masticatory problems, and alterations in the taste and speech. In this paper we present a case of true macroglossia diagnosed in a female patient, 26 years, melanoderma, no family history of disease, with a history of relapse of orthodontic treatment for correction of open bite, loss of the lower central incisors, and complaint of difficulty in phonation. The patient was submitted to glossectomy under general anesthesia using the “keyhole” technique, with objective to provide reduction of the lingual length and width. The patient developed with good repair, without taste and motor alterations and discrete paresthesia at the apex of the tongue.

## 1. Introduction

Macroglossia is an uncommon condition that can lead to several alterations like dental-muscle-skeletal deformities, orthodontic treatment instability, masticatory, and breathing and phonation problems, characterized by increased size of the tongue, can be caused by congenital malformations or acquired diseases. The most common causes are muscle hypertrophy and congenital vascular malformations, such as lymphangioma and hemangioma, they are also characteristics found in the Beckwith-Wiedemann syndrome, and can be present in the Down syndrome [1–14]. It can be acquired as a result of amyloidosis, myxedema, angioedema, and macromegalia [1, 5, 14–19]. The tongue can also be normal in size but can seem increased when compared with adjacent structures because of anteroposterior mandible or maxillary transverse deficiency or also due to cysts, tumors, and tonsillar hyperplasia that can move up and out the tongue. This last condition is called pseudomacroglossia and must be differentiated from true macroglossia, because its correction is achieved by treating the primary disease [20].

Accurate diagnosis of true macroglossia is obtained through the signs and symptoms of this alteration, which is of fundamental importance for the correct indication for surgical treatment, in order to restore proper function and provide stability for orthodontic treatment [21].

The surgical treatment indicated for the true macroglossia is the reductive glossectomy. Several techniques have been proposed in the literature to enable the reduction of the tongue. Peripheral incisions with marginal resection of tissue have as complications hipomobility and change in the form of the tongue that becomes globular [2, 5, 16, 20, 22]. Incisions V-shaped positioned in the midline of the tongue are effective in reducing the length but are ineffective in reducing the width of the tongue [3, 23, 24]. Elliptical incision positioned in the midline without reaching the apex of the tongue contributes to reducing the width with little influence on its length [25]. Incisions in the form of keyhole combine characteristics of elliptical and V-shaped incisions and are indicated when the reduction of the width and length of the tongue are desirable and its design can be changed according to the specific needs of each case [9, 26, 27].

This paper aims to present a case of true macroglossia treated surgically using the keyhole technique.

## 2. Case Report

**Figure 1:**
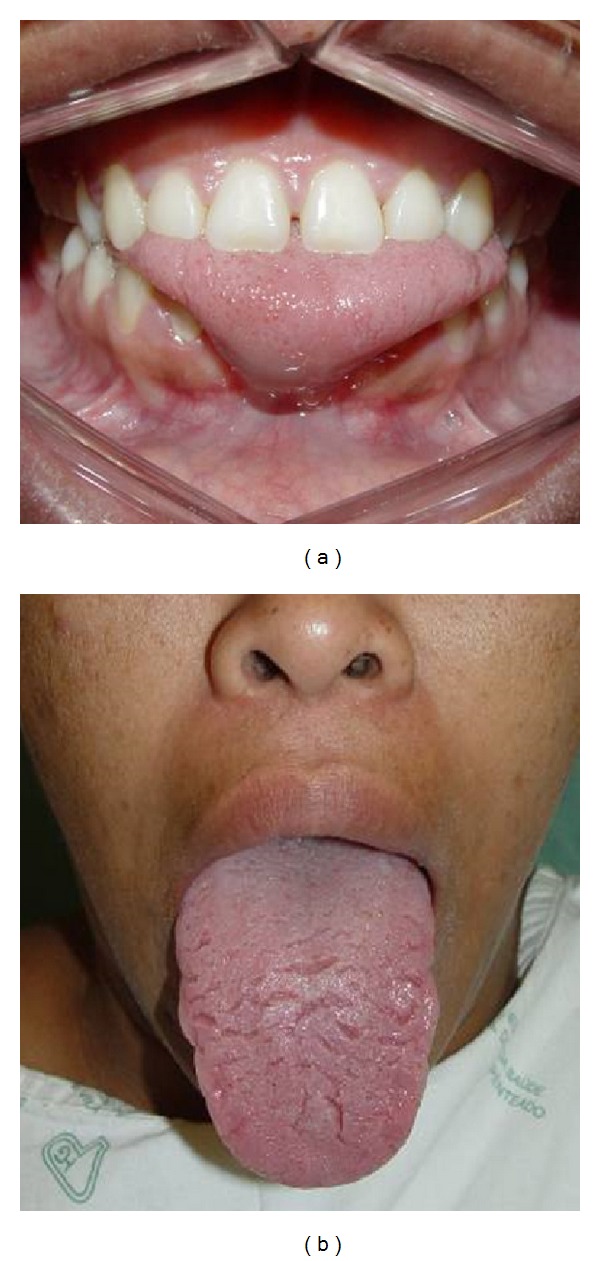
The tongue was increased both in length and in width, with anterior open bite with interposition of the tongue and loss of 31 and 41 teeth.

Female patient, 26 years, melanoderma, was referred by the Department of phonoaudiology with complain of difficulty in speech and history of orthodontic treatment for correction of anterior open bite that resulted in complete recurrence. No history of systemic diseases or drug allergies or family history of her disease. On physical examination, the tongue was increased both in length and in width, with anterior open bite and interposition of the tongue, impression of the lingual surface of mandible molars on the edges of the tongue, loss of the tooth 31 and 41, occlusion in Class I of Angle, reversion Spee curve in the mandible and marked in the maxilla (Figures 1(a) and 1(b)), with diagnosis of true macroglossia requiring multidisciplinary treatment involving surgery, 1 orthodontics, and fonoaudiology.

**Figure 2:**
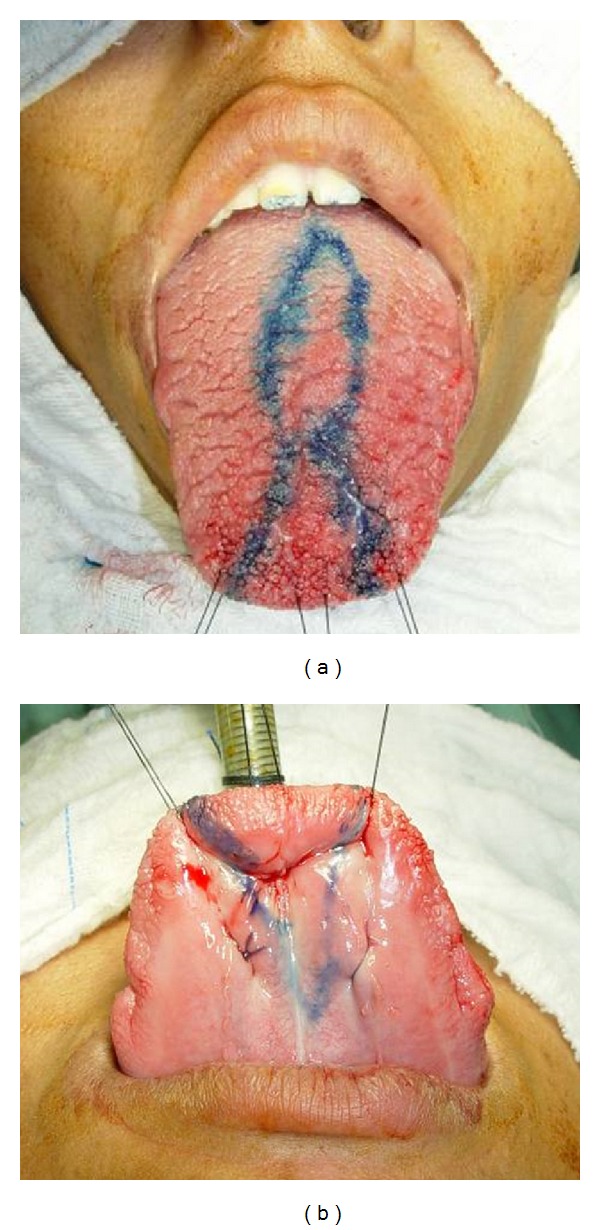
The tongue was pulled out of the oral cavity through three repairs with nylon suture 3-0 fixed to the surgical field to maintain the symmetry between the sides and facilitate the demarcation of the incisions, which was performed which methylene blue.

**Figure 3:**
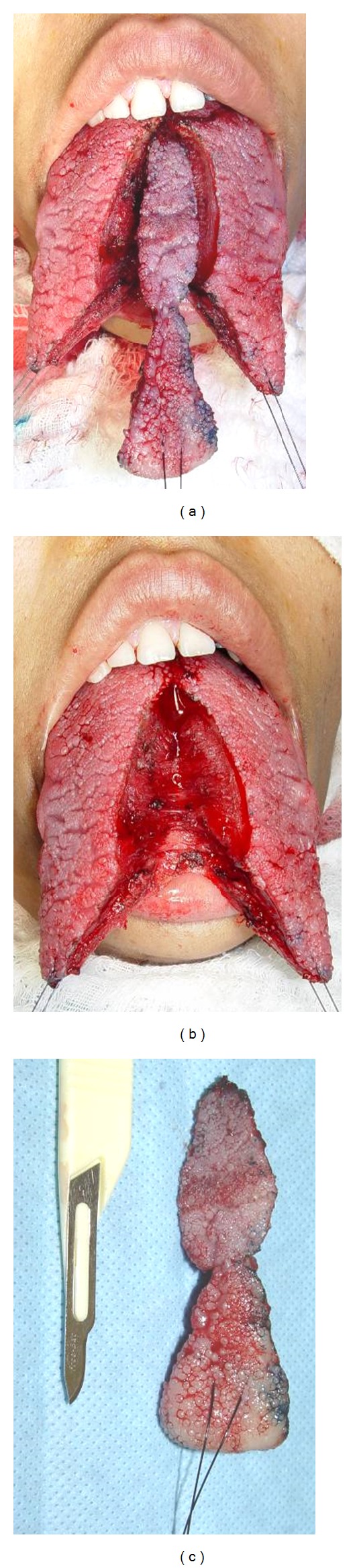
A partial thickness elliptical wedge incision starting at the midline and 4 mm distance from tongue taste buds using electrocautery, the dorsum, and incisions on belly anterior tongue were united resulting in a full-thickness flap, proceeding the excision of excess tissue.

**Figure 4:**
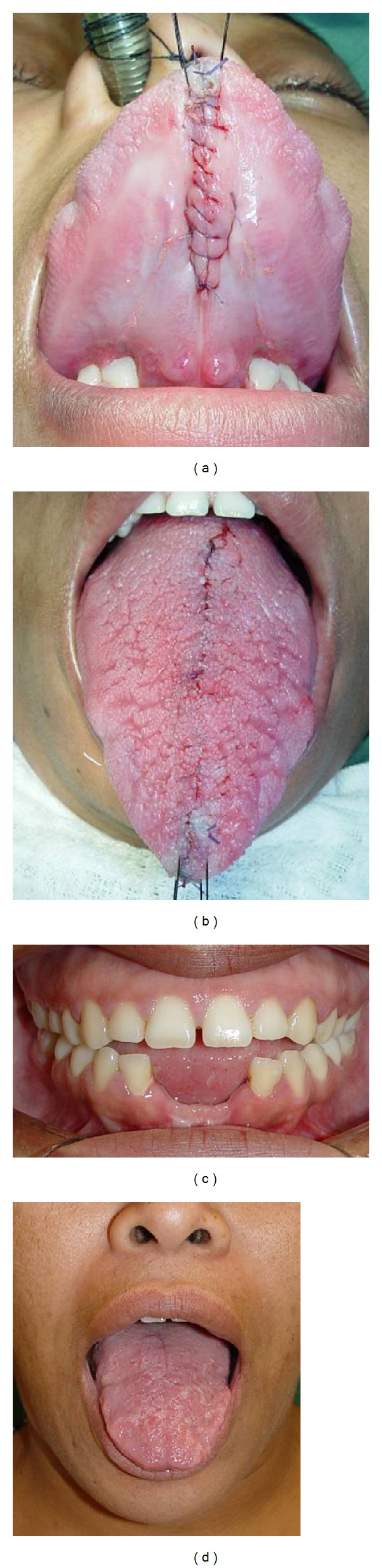
The suture was made by planes with polyglactin 910 suture 3-0, good tissue repair, perfect symmetry, and no tongue interposition.

The technique chosen for reduction glossectomy was the “keyhole” technique with the goal of reducing the tongue in width and length. The patient underwent general anesthesia nasotracheal intubation, and the tongue was pulled out of the oral cavity through three repairs with nylon suture 3-0 fixed to the surgical field to maintain the symmetry between the sides and facilitate the demarcation of the incisions, which was performed with methylene blue (Figures 2(a) and 2(b)). After infiltration of lidocaine 2% with epinephrine 1 : 200.000, a partial thickness elliptical wedge incision was made in the tongue dorsum, starting at the midline and at 4 mm away from tongue taste buds, as well as, incisions on dorsum and belly anterior tongue using electrocautery, which were united resulting in a full-thickness flap, proceeding the excision of excess tissue (Figures 3(a), 3(b), and 3(c)). The suture was made by planes with polyglactin 910 suture 3-0. In the immediate postoperative period there was slight swelling in the floor of the mouth and on belly anterior tongue, and discrete lingual hypoesthesia, and it evolved after a week without taste or motor alteration and with good tissue repair, perfect symmetry, and no tongue interposition. The patient was referred to orthodontic and fonoaudiology treatment (Figures 4(a), 4(b), 4(c), and 4(d)).

## 3. Discussion

To determine the real need of surgical treatment is of fundamental importance to define the signs and symptoms of true macroglossia. The diagnosis must be based on clinical findings [20].

In cases of true macroglossia, as presented, in the literature several reductive glossectomy techniques are suggested. Each technique has a purpose in accordance with the clinical aspect.

In the presented case the keyhole technique was used which is indicated for reduction of length and width of the tongue, preserving the vasculonervous bundle and with few reports of sensory and functional deficits [9, 25, 28]. Sensitivity, movement, and taste when modified are resolved spontaneously within a few weeks [10, 29] and these observations from the literature were confirmed in our experience.

## 4. Conclusion

Surgical reduction of the tongue is an uncommon procedure, being indicated in specific cases; the keyhole technique allows anterior and median partial resection, technically simple, minimal morbidity, and good functional result.
